# Studies on the Reaction of Dietary Methylglyoxal and Creatine during Simulated Gastrointestinal Digestion and in Human Volunteers

**DOI:** 10.3390/nu14173598

**Published:** 2022-08-31

**Authors:** Stephanie Treibmann, Julia Groß, Susann Pätzold, Thomas Henle

**Affiliations:** Chair of Food Chemistry, Technische Universität Dresden, D-01062 Dresden, Germany

**Keywords:** digestion, gastrointestinal tract, 1,2-dicarbonyl compounds, methylglyoxal, Maillard reaction, glycation, creatine, urine, metabolism

## Abstract

The reactive 1,2-dicarbonyl compound methylglyoxal (MGO) is consumed with food and its concentrations decrease during digestion. In the present paper, the reaction of MGO with creatine, arginine, and lysine during simulated digestion, and its reaction with creatine during the digestion in human volunteers, was studied. Therefore, simulated digestion experiments with a gastric and an intestinal phase were performed. Additionally, an intervention study with 12 subjects consuming MGO-containing Manuka honey and creatine simultaneously or separately was conducted. Derivatization with o-phenylenediamine and HPLC–UV was used to measure MGO, while creatine and glycated amino compounds were analyzed via HPLC–MS/MS. We show that MGO quickly reacts with creatine and arginine, but not lysine, during simulated digestion. Creatine reacts with 56% of MGO to form the hydroimidazolone MG-HCr, and arginine reacted with 4% of MGO to form the hydroimidazolone MG-H1. In the intervention study, urinary MG-HCr excretion is higher in subjects who consumed MGO and creatine simultaneously compared to subjects who ingested the substances separately. This demonstrates that the 1,2-dicarbonyl compound MGO reacts with amino compounds during human digestion, and glycated adducts are formed. These contribute to dietary glycation products consumed, and should be considered in studies investigating their physiological consequences.

## 1. Introduction

Methylglyoxal (MGO) is a dicarbonyl compound formed during food processing and in vivo. It is an intermediate of caramelization and glycation reactions (Maillard reaction), and it is also formed as a byproduct of glycolysis (ca. 3 mmol/d) [1,2,3,4]. MGO is highly reactive towards proteins and DNA, and can impair the functionality of the derivatized protein or DNA molecule. Increased formation and/or decreased metabolization of MGO in vivo, such as in patients with diabetes or uremia, contributes to “dicarbonyl stress” [5]. Therefore, research was conducted on ways to decrease MGO concentrations in vivo. Scavengers such as aminoguanidine, creatine, flavonoids, and polyphenols were examined in model systems [6,7], in foods [8,9], and human intervention studies [10,11]. Additionally to the in vivo formation, humans also consume around 0.04 [12] to 0.3 mmol [13] MGO per day with food. However, it was found that dietary MGO does not increase the concentrations of MGO or its metabolite d-lactate in urine [14]. This points towards a poor bioavailability of MGO and possible reactions during digestion, which are shown in many studies. While MGO is mostly stable during a simulated gastric stage at pH 2, it reacts rapidly during the duodenal or intestinal stage [14,15,16,17,18]. In most studies using simulated digestion, only 10–50% of MGO is left after 6 to 8 h. Concentrations of other dicarbonyl compounds such as 3-deoxyglucosone also decrease during digestion [17,19].

The decrease in the amount of MGO is not merely a pH effect, since it is stable in incubations at 37 °C and pH 2 and pH 8 for several hours [14]. However, its concentrations decrease when enzymes are added to the incubation, which suggests glycation reactions of MGO with amino side chains during the duodenal or intestinal stage. There are few studies on the formation of MGO-derived and other glycation reaction products during simulated digestion. For example, during the simulated digestion of model incubations and of biscuits where the decrease in MGO is shown, masses of adducts of MGO and 3-deoxyglucosone with lysine, cysteine, arginine, and histidine are observed with high-resolution mass spectrometry [17,18]. However, to the best of our knowledge, there are no quantitative data published on the formation of glycated amino acids during the digestion of MGO. Some other glycation reaction products formed during simulated digestion were quantified. The Amadori compound fructosyllysine, measured as furosine, was quantitated after simulated digestion of glucose and gluten where it is formed in concentrations of up to 78.9 nmol/mg protein [17], which is a higher protein modification than found in foods such as bakery products (0.8–23.6 nmol furosine/mg protein) [20]. In simulated digestions of biscuits, the MGO-derived hydroimidazolone of arginine (MG-H1) increases by more than 400% [21]. This quantitative data underlines that the formation of Maillard reaction products during digestion might contribute significantly to the amount of Maillard reaction products absorbed.

Knowledge about the reactions of MGO during digestion is important to understand natural processes, especially because it can also react with gastric and intestinal cells [22]. Cytotoxic effects of MGO on epithelial cells were discussed, but seemed to only occur at concentrations higher than what is normally achieved by diet [15,23]. Dicarbonyl compounds might also increase the secretion of pro-inflammatory cytokines such as interleukin-8 [24] and, furthermore, may have an impact on the gut microbiome [25]. Due to those potentially adverse effects, some groups have quantitated the MGO-scavenging capacity of amino acids and foods [18,26]. In simulated digestion experiments, cysteine and glycine, but not lysine, glutamine, asparagine, and glutamate, could scavenge MGO [18]. Different foods containing arginine, creatine, thiol groups, and flavonoids scavenged MGO under simulated intestinal conditions [26]. However, to the best of our knowledge, no in vivo studies dealing with the reactions of MGO during digestion have been performed yet.

The aim of this study, therefore, is to quantitatively describe the reactions of MGO with creatine, arginine, and lysine, and the formation of the respective adducts during simulated digestion. Additionally, we want to investigate the reaction of MGO and creatine during digestion in vivo via a human intervention study.

## 2. Materials and Methods

### 2.1. Chemicals 

Creatinine, lysine monohydrate, and urea were purchased from Merck (Darmstadt, Germany); HPLC grade methanol, and sodium chloride from VWR ProLabo (Leuven, Belgium); creatine monohydrate from Thermo Fisher Scientific (Waltham, MA, USA); and formic acid from Roth (Karslruhe, Germany). Bile extract (porcine, lyophilized, B8631), 2-methylquinoxaline, 40% MGO, *o*-phenylenediamine, mucin (from porcine stomach, type III, M1778), pancreatin (from porcine pancreas, P1500), pepsin (from porcine gastric mucosa, P7000), and trypsin (from porcine pancreas, type IX-S, To3o3) from Sigma-Aldrich were employed (Steinheim, Germany) and amino guanidine from Cayman Chemical Company (Ann Arbor, MI, USA). Isotopically labeled ^2^D_3_-creatine and ^2^D_3_-creatinine were obtained from Cambridge Isotope Laboratories (Loughborough, UK) and disodium hydrogen phosphate, sodium dihydrogen phosphate, ammonium formate, calcium chloride dihydrate, potassium chloride, potassium phosphate monobasic, and sodium carbonate from Grüssing (Filsum, Germany). Arginine was purchased from FLUKA (Buchs, Switzerland) and LC–MS grade acetonitrile from Th. Geyer (Renningen, Germany). Water for solutions, buffers, and HPLC–MS/MS was obtained from a Bi 18 E double distillation system (QCS, Maintal, Germany). The substances MGO-derived hydroimidazolone of creatine (MG-HCr) [6], D_3_-MG-HCr [6], *N^ε^*-carboxyethyllysine (CEL) [27], ^13^C_3_-CEL [27], MG-H1 [27], and ^13^C_6_-MG-H1 [27] were synthesized and characterized in our laboratory, according to the literature stated. The substances met the spectroscopic and chromatographic characteristics published in the respective protocols.

### 2.2. Digestion Experiments

Simulated in vitro digestions of MGO were performed as described in a previous study [14]. For the gastric stage, 50 µL of either 300 mM creatine monohydrate, arginine, or lysine monohydrate, or 50 µL of water was added to 4 mL of synthetic gastric solution (2.9 g/L sodium chloride, 0.7 g/L potassium chloride, 0.27 g/L potassium phosphate monobasic) and the pH was adjusted to 2.0 with 3 N HCl. For simulated digestions with digestive enzymes, 0.63 mg/mL pepsin and 1.88 mg/mL mucin were dissolved in the gastric solution prior to the experiment. After adding 50 µL of 30 mM MGO, pH was adjusted again to 2.0 with 3 N HCl. After stirring at 37 °C for 2 h, 3 mL of synthetic intestine solution (0.3 g/L potassium chloride, 0.5 g/L calcium chloride dihydrate, 0.3 g/L urea, 0.2 g/L sodium carbonate) was added and the pH was adjusted to 7.5 with solid sodium carbonate. For simulated digestions with digestive enzymes, 9.0 mg/mL bile extract, 0.33 mg/mL trypsin, and 8.0 mg/mL pancreatin were dissolved in the intestinal solution prior to adding it to the samples. The mixture was stirred at 37 °C for 6 h, with hourly corrections of the pH value to 7.5. Samples (500 µL) were withdrawn after 10 and 120 min under simulated stomach conditions and after 10, 60, 180, and 360 min under simulated intestinal conditions. An aliquot (250 µL) of the samples was subjected to RP–HPLC–UV analysis of MGO. For analysis of MG-HCr and glycated amino acids, 250 μL of the sample was added to 25 µL of 18.5 mM amino guanidine (to stop derivatization with MGO), 750 μL of solvent mixture (86% of 0.1% formic acid in acetonitrile, 14% of 100 mM ammonium formate in water), and 10 µL of internal standard (5.40 µg/mL D_3_-MG-HCr, 62.5 µg/mL D_3_-creatine, 62.5 µg/mL D_3_-creatinine, 3.75 µg/mL ^13^C_3_-CEL, 0.75 µg/mL ^13^C_6_ MG-H1). The samples were stored at −18 °C for 30 min for protein precipitation, centrifuged (10 min, 10,600× *g*) and subjected to HPLC–MS/MS.

### 2.3. Human Intervention Study

The Ethics Committee of Technische Universität Dresden approved the study (reference: EK 503122018). Written consent was obtained from each participant. All 12 participants were healthy adults (ages 22–30 years, 11 women and 1 man, body mass index 18.8–25.3). Prior to the study, participants were asked to follow a vegan diet for 6 days. During the duration of the study (3 days), subjects followed a vegan diet and collected 24 h urine samples from 8:00 AM until 8:00 AM the following day. Food questionnaires were filled out on all study days. On day 2, both groups had intervention meals. Group 1 (6 subjects) had 2.5 g of creatine mono hydrate, 65 g of Manuka honey (660 mg/kg MGO), and 3 crispbreads at 9 a.m., while group 2 had 2.5 g of creatine monohydrate at 9 a.m. and 65 g of Manuka honey (660 mg/kg MGO), and 3 crisp breads at 3 p.m. Creatine monohydrate was purchased from Fitmart (Rellingen, Germany), Manuka honey from Berringa (Melbourne, Australia), and crispbread from a local retailer. Aliquots of 24 h urine samples were frozen at −18 °C until analysis. Creatine, creatinine, and MG-HCr in urine were analyzed as described before [28]. Samples were thawed and centrifuged (4 °C, 10 min, 10,600× *g*). An aliquot (250 µL) was added to 750 µL of solvent mixture (86% of 0.1% formic acid in acetonitrile, 14% of 100 mM ammonium formate in water) and 10 µL of internal standard (5.40 µg/mL D_3_-MG-HCr, 62.5 µg/mL D_3_-creatine, 62.5 µg/mL D_3_-creatinine, 3.75 µg/mL ^13^C_3_-CEL, 0.75 µg/mL ^13^C_6_ MG-H1). After 30 min at 4 °C, samples were centrifuged again (4 °C, 10 min, 10,600× *g*) and subjected to HPLC–DAD–ESI–MS/MS.

### 2.4. High-Pressure Liquid Chromatography with UV Detection (HPLC–UV) of MGO after Derivatization to Quinoxalines

Dicarbonyl compounds were analyzed as described previously [13]. For protein precipitation, 250 μL of the sample was added to 500 μL of methanol and stored at −18 °C for 30 min. After centrifugation (10 min, 10,600× *g*), 150 μL of 0.5 M phosphate buffer (pH 6.5) and 150 μL of 0.2% *o*-phenylenediamine were added to 500 μL of the supernatant. The mixture was kept in the dark for 16 h and centrifuged (10 min, 10,600× *g*). Analysis was carried out as published previously on a RP–HPLC system with UV detection [29]. External calibration was performed with the quinoxaline of MGO, 2-methylquinoxaline.

### 2.5. High-Pressure Liquid Chromatography with Tandem Mass Spectrometric (HPLC–MS/MS) Detection

Creatine, creatinine, MG-HCr, and the glycated amino acids CEL and MG-H1 were analyzed as described before [28] on a high-pressure gradient system 1200 Series (Agilent Technologies, Böblingen, Germany), consisting of a binary pump, an online degasser, an autosampler, a column oven, and a diode array detector. Chromatographic separation was performed on an Intrada Amino Acid column (50 × 2 mm, 3 μm, Imtakt (USA)) at 35 °C with 0.1% formic acid in acetonitrile (solvent A) and 100 mM ammonium formate in water (solvent B) as the mobile phases. Analysis was conducted using a flow rate of 0.4 mL/min and an injection volume of 8 μL. The solvent composition started at 14% B for 3 min, was increased to 100% B within 7 min, decreased to 14% B within 2 min, and held at 14% B for 4 min. The HPLC was coupled to a mass spectrometer 6410 Triple Quad (Agilent, Böblingen, Germany) working with electrospray ionization and a capillary voltage of 4000 V in the positive mode and a source temperature of 350 °C. The nebulizing gas nitrogen (nitrogen generator 5183-2003, Agilent) had a pressure of 35 psi and a flow rate of 11 L/min. Conditions for the multiple reaction monitoring mode (MRM) are listed in Table 1. Analytes were quantitated by isotope dilution analysis.

### 2.6. Statistical Treatment

All samples were analyzed in duplicate. Normal distribution was tested with the Shapiro–Wilk test (*p* = 0.05). Comparison of means was conducted with analysis of variance (ANOVA) and Scheffe post hoc test for normal distributed data.

## 3. Results

### 3.1. Methylglyoxal (MGO) Reacts with Lysine, Arginine, and Creatine during Simulated Digestion

**Figure 1:**
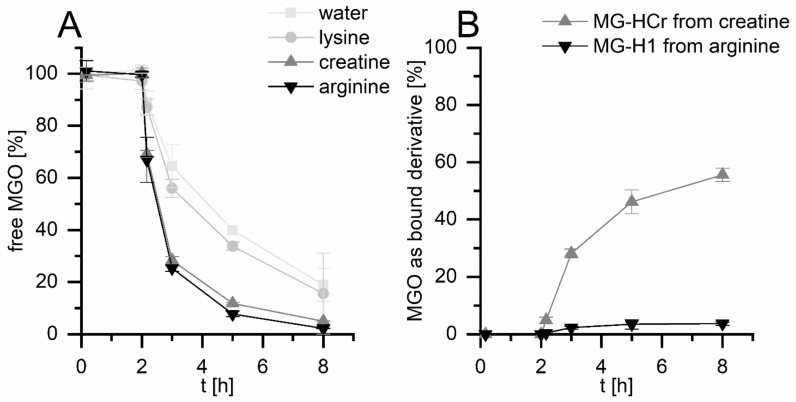
Simulated digestion experiments (2 h gastric stage, 6 h intestinal stage) of MGO with and without arginine, lysine, and creatine (molar ratio 1:10) in the presence of added digestive enzymes/bile. Decrease in unbound MGO measured with HPLC–UV (**A**,**B**); formation of bound MGO in the form of MG-HCr (from creatine) and MG-H1 (from arginine) measured with HPLC–ESI–MS/MS (**B**).

The first aim of this study was to obtain quantitative data about the formation of MGO-derived glycation compounds during simulated digestion. Therefore, simulated digestion experiments of MGO with the amino compounds creatine, arginine, and lysine were conducted. The digestion consisted of a gastric phase at pH 2 for 2 h and an intestinal phase at pH 7.5 for 6 h. The synthetic digestion solutions consisted of the respective electrolytes, and all experiments were run with and without added digestive enzymes and bile to investigate the influence of those added proteins. The molar ratio of amino compounds to MGO was 10:1. MGO was measured with HPLC–UV after deproteinization with methanol and derivatization with *o*-phenylenediamine as described before [13]. Comparable to the literature, MGO is mostly stable in experiments without added enzymes and amino compounds (Figure S1A Supporting Information), and decreases to ca. 20% when digestive enzymes are added (Figure 1A) [14]. Lysine only has a minor influence on the MGO concentrations. However, both creatine and arginine decrease the MGO concentrations to 8% and 3%, respectively, in the presence and absence of added enzymes. This shows that creatine and arginine, but not lysine, can reduce MGO concentrations during digestion, which confirms what is stated in the literature [7,18,26].

The formation of glycated amino compounds was measured via HPLC–MS/MS and isotope dilution analysis, as described previously [28]. One major MGO adduct was selected for each digested substance. Therefore, *N^ε^*-carboxyethyllysine (CEL) was analyzed as a lysine derivative, *N^δ^* -(5-hydro-5-methyl- 4-imidazolon-2-yl)-ornithine (MG-H1) as an arginine derivative, and *N*-(4-methyl-5-oxo-1-imidazolin-2-yl)sarcosine (MG-HCr) as a creatine derivative. As shown in Figure 1B, no glycated amino compound formation is measurable after the gastric stage, while the concentrations increase during the intestinal stage. In the experiments with digestive enzymes, 56% of MGO reacts with creatine to form MG-HCr, 4% of MGO reacts with arginine to form MG-H1, and 0.06% of MGO reacts with lysine to form CEL. In experiments without digestive enzymes, the formation of glycated amino compounds is only slightly higher (Figure S1B Supporting Information). This shows that the formation of glycated creatine and arginine is not altered substantially by additional protein.

The concentrations of MG-HCr, CEL, and MG-H1 formed are comparable to the literature. In model incubations of creatine and MGO (1:1) at pH 7.4 and 37 °C, ca. 40% of MGO reacts to form MG-HCr after 24 h and 75% after 24 h [6]. In model incubations of *N^α^-tert-*butoxycarbonyl-arginine and MGO (1:1) at pH 7.4 for 6 h, ca. 10% of MGO reacts to form MG-H1, ca. 10% forms the tetrahydropyrimidine *N^δ^*-(4-carboxy-4,6-dimethyl-5,6-dihydroxy-1,4,5,6-tetrahydropyrimidine-2-yl)-*L*-ornithine, ca. 6% forms argpyrimidine, ca. 3% forms *N^ε^*-carboxyethylarginine, and ca. 1% forms MG-H3 [30]. The formation of those other products might explain the discrepancy between the decrease in MGO and the formation of MG-H1 from arginine. In model incubations of RNase and MGO (lysine:MGO 1:1), 0.5% CEL forms after 10 h at pH 7.4 and 37 °C [31]. In conclusion, arginine and creatine, but not lysine, quickly react with MGO and form glycated amino compounds during digestion. Creatine mainly forms MG-HCr, while arginine has a wider product spectrum, and MG-H1 only accounts for a smaller part of the arginine–MGO reactions.

### 3.2. Reaction of MGO and Creatine during Simultaneous Digestion in Human Volunteers

Due to difficulties in acquiring the content of the digestive tract of humans, the fast reactions of MGO with different compounds in all matrices, and the high in vivo turnover of MGO, reactions of MGO during digestion in vivo can hardly be analyzed. Therefore, the reaction product must be measured. In order to prove a reaction of MGO and formation of possible adducts in vivo, the reaction of creatine and MGO was studied, because this reaction has a narrow product spectrum and a high recovery rate of MGO as MG-HCr with over 50%. We, therefore, conducted a study in which participants consumed the dietary supplement creatine monohydrate and Manuka honey, which contains MGO.

In the study, 12 healthy volunteers were divided into two groups (6 subjects per group). Both groups had a vegan diet virtually free of creatine for 6 days prior to the study as a washout period [28] and throughout the whole study. During the three day study, subjects collected 24 h urine samples and had an intervention meal on day two. To analyze the reaction of creatine and MGO, group 1 (simultaneous ingestion group, SIM group) consumed 2.5 g of creatine monohydrate, 65 g of Manuka honey (containing 43 mg MGO), and 3 crispbreads at 9 a.m. As a control, group 2 (separate ingestion group, SEP group) ingested 2.5 g of creatine monohydrate at 9 a.m. and 65 g of Manuka honey (containing 43 mg MGO) and 3 crisp breads at 3 p.m. Crisp bread was added to make the honey consumption more pleasant, and to prolong the presence of MGO in the digestive tract. The separate ingestion group had the same meal components to ensure that the effect of the intervention is due to the reaction of the consumed components during digestion. Creatine and glycated amino compounds in urine samples were analyzed via HPLC–MS/MS, as described before [28].

**Figure 2:**
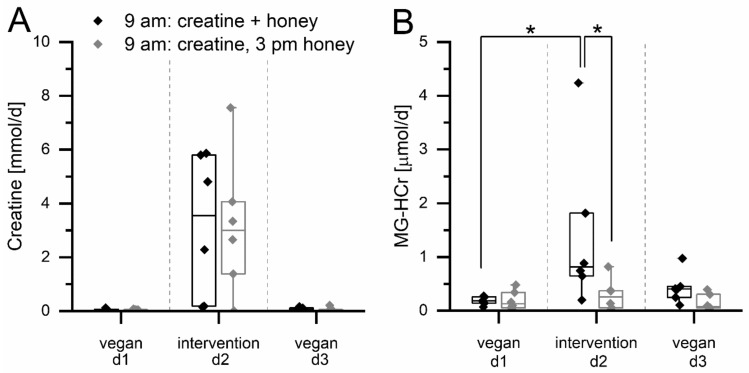
Excretion of creatine (**A**) and MG-HCr (**B**) in 24 h urine samples of 12 volunteers during a three day study with an intervention on day 2. Intervention: Simultaneous ingestion group (black): 2.5 g of creatine monohydrate + 65 g of Manuka honey (43 mg MGO) + 3 crispbreads at 9 a.m., separate ingestion group (grey): 2.5 g of creatine monohydrate at 9 a.m. and 65 g of Manuka honey (43 mg MGO) and 3 crispbreads at 3 p.m. Significance: * *p* < 0.05.

Since both groups ingested creatine on the intervention day, urinary creatine excretion increases from 0.06 ± 0.03 µmol/d (SIM group) and 0.04 ± 0.02 µmol/d (SEP group) to 3.2 ± 2.7 mmol/d (SIM group) and 3.2 ± 2.6 mmol/d (SEP group, see Figure 2A). This shows that between 1% and 45% of ingested creatine (mean 19%) is excreted with urine. Creatinine excretion does not change significantly during the study (Figure S2 Supporting Information). As shown in Figure 2A, the creatine excretion does not differ significantly between the groups. This shows that both groups should have absorbed similar amounts of creatine. Thus, if there is some in vivo MG-HCr formation from the supplemented creatine, it should not be different between the groups.

On the other hand, the excretion of MG-HCr increases from 0.18 ± 0.08 µmol/d (SIM group) and 0.19 ± 0.19 µmol/d (SEP group) to 1.4 ± 1.5 µmol/d (SIM group) and 0.29 ± 0.30 µmol/d (SEP group, Figure 2B). The excretion of MG-HCr of the simultaneous ingestion group is significantly higher on the intervention day than on the vegetarian day (*p* < 0.05, two-way ANOVA with repeated measures, Scheffe post hoc test), whereas for the separate ingestion group, no significant increase is observed. Additionally, MG-HCr excretion of the simultaneous ingestion group on the intervention day is also significantly higher than of the separate ingestion group on the intervention day (*p* < 0.05, two-way ANOVA with repeated measures, Scheffe post hoc test).

The simultaneous ingestion group excrete 0.1 mg more MG-HCr with urine compared to the separate ingestion group, which accounts for 0.24% of MGO consumed with Manuka honey.

## 4. Discussion

In this study, we show that MGO reacts with creatine during digestion to form MG-HCr not only during simulated digestion, but also in human volunteers. In simulated digestion experiments, creatine reacts with 56% of MGO to form the glycation compound MG-HCr, arginine forms MG-H1 with 4% of the added MGO, and lysine only reacts with 0.06% of MGO to form CEL. In the human intervention study, volunteers who consumed creatine and MGO together excrete ca. 1.1 µmol/d MG-HCr more compared to volunteers who consumed it separately.

It cannot be ruled out that additional reactions in epithelial cells or plasma after absorption of both compounds may occur. However, the formation of MG-HCr is highly likely to happen during digestion. The reasons are (a) MG-HCr excretion does not increase when the substances are given separately. Since both MGO and creatine are abundant in vivo, there should been an increase in the SEP group if the reaction happens in cells or plasma. (B) MGO is likely to be quickly metabolized after entering intestinal epithelial cells, because they express glyoxalase 1, which detoxifies MGO [32]. Additionally, MGO consumption does not lead to an increase in the excretion of MGO or its metabolites [14]. Hence, MGO concentrations and, thereby its ability to react with creatine, should be highest in the intestinal lumen during digestion. (C) The reaction of MGO and creatine is also quite fast [6], thus, most of the MG-HCr formation should happen during digestion.

This demonstrates that the 1,2-dicarbonyl compound MGO reacts with amino compounds during human digestion and glycated adducts are formed. The excreted MG-HCr accounts for 0.24% of the consumed MGO. The recovery of MGO as MG-HCr in urine is thereby much lower than after simulated digestion (56%). Reasons for the lower recovery might be the low bioavailability of MG-HCr (ca. 54%) [28], reactions of MGO with other substances during digestion such as cell membranes or food protein, and the rapid absorption of creatine [33].

These observations have two main consequences. On the one hand, MGO concentrations decrease during digestion, especially with added creatine. Creatine, therefore, could contribute to a “natural” protection from the MGO-derivatization of protein or DNA of epithelial cells, as is discussed by other groups [26]. The findings also offer an explanation for the poor bioavailability of MGO [14]. On the other hand, reactions of MGO during digestion form glycated amino compounds such as MG-HCr, MG-H1, and CEL. Those glycation compounds are also part of the diet and are discussed to be a risk factor for chronic diseases [34]. However, this possible pathophysiological role of glycation compounds is highly controversial [35]. Estimations of the daily consumption of MGO range from 0.04 [12] to 0.3 mmol [13]. Therefore, 0.04 to 0.3 mmol of MGO-derived glycated amino compounds could potentially form during digestion. Compared to that, the daily consumption of MGO-derived glycated amino compounds with the diet is 0.07–0.13 mmol MG-H1 [36], 0.007–0.014 mmol CEL [36], and ca. 0.006 mmol MG-HCr [9]. Hence, glycation compounds formed during digestion could considerably contribute to the total amount of glycated amino compounds absorbed. These digestive glycated amino compounds should be considered when studying the effects of dietary glycated compounds on health and disease. Glycated products formed during digestion might explain the discrepancy between different studies researching the physiological effects of dietary glycated products [35].

Further research could focus on the reasons for the lower recovery rate of MGO as MG-HCr after digestion in human volunteers compared to simulated digestion, on the glycation reactions of other compounds, and on physiological implications of glycation reactions during digestion.

## Figures and Tables

**Table 1:** Transitions recorded during MRM measurement.

	Transition	Fragmentor Voltage [V]	Collision Energy [eV]	Dwell Time [ms]	Q/q ^1^
MG-HCr	186 → 87	110	20	180	Q
	186 → 44	110	25	180	q
D_3_-MG-HCr	189 → 90	110	20	130	Q
	189 → 44	110	25	130	q
CEL	219 → 130	100	10	70	q
	219 → 84	100	20	70	Q
^13^C_3_-CEL	222 → 130	90	10	70	q
	222 → 84	90	20	70	Q
Creatine	132 → 90	75	10	60	Q
	132 → 44	75	20	60	q
D_3_-Creatine	135 → 93	90	10	60	Q
	135 → 47	90	20	60	q
Creatinine	114 → 86	105	10	50	Q
	114 → 44	105	14	50	q
D_3_-Creatinine	117 → 89	105	10	50	Q
	117 → 47	105	14	50	q
MG-H1	229 → 166	75	10	120	q
	229 → 114	75	10	120	Q
^13^C_6_-MG-H1	235 → 171	120	10	120	q
	235 → 115	120	10	120	Q

^1^ Q, transition used for quantitation; q, transition used for the confirmation of the presence of the analyte.

## Data Availability

The data are available on request from the authors.

## Supplementary Materials

The following supporting information can be downloaded at: https://www.mdpi.com/article/10.3390/nu14173598/s1, Figure S1: Simulated digestion experiments in the absence of digestive enzymes and bile; Figure S2: Daily excretion of creatinine via urine.
