# Supplementary material for: Studies on the Reaction of Dietary Methylglyoxal and Creatine during Simulated Gastrointestinal Digestion and in Human Volunteers
Source: Nutrients. 2022 Aug 31;14(17):3598. doi: 10.3390/nu14173598 (PMC9460645; doi:10.3390/nu14173598)
Supplement: Supplementary file 1 [file nutrients-14-03598-s001.zip › nutrients-1888525-supplementary.pdf]

information

Supplementary Materials

Studies on the Reaction of Dietary Methylglyoxal and Creatine During Simulated Gastrointestinal Digestion and in Human Volunteers

Stephanie Treibmann [1,*], Julia Groß [1], Susann Pätzold [1] and Thomas Henle [1,*]

[1] Chair of Food Chemistry, Technische Universität Dresden, D-01062 Dresden, Germany
* Correspondence: Stephanie.treibmann@tu-dresden.de, Thomas.henle@tu-dresden.de; Tel.: +49-351-463-34647

List of Supporting Information

Figure S1. Simulated digestion experiments in the absence of digestive enzymes and bile.

Figure S2. Daily excretion of creatinine via urine.

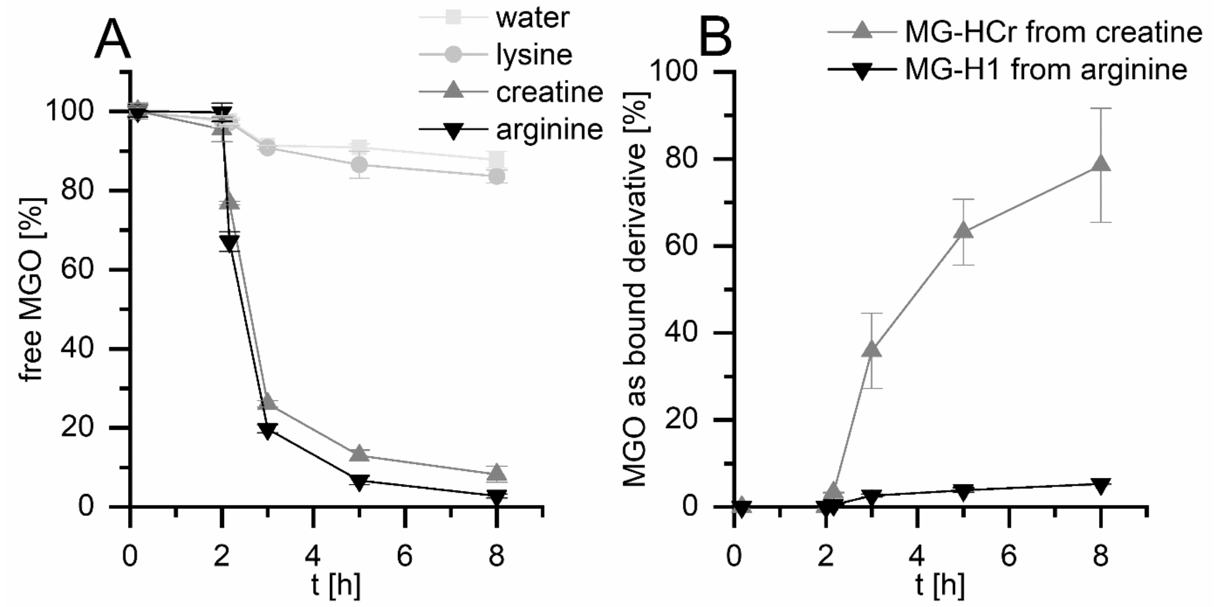

Figure S1. Simulated digestion experiments in the absence of digestive enzymes and bile. Simulated digestion experiments (2 h gastric stage, 6 h intestinal stage) of MGO with and without arginine, lysine, and creatine (molar ratio 1:10) in the absence of added digestive enzymes/bile. Decrease of unbound MGO measured with HPLC-UV (A) and (B) formation of bound MGO in the form of MG-HCr (from creatine) and MG-H1 (from arginine) measured with HPLC-ESI-MS/MS (B).

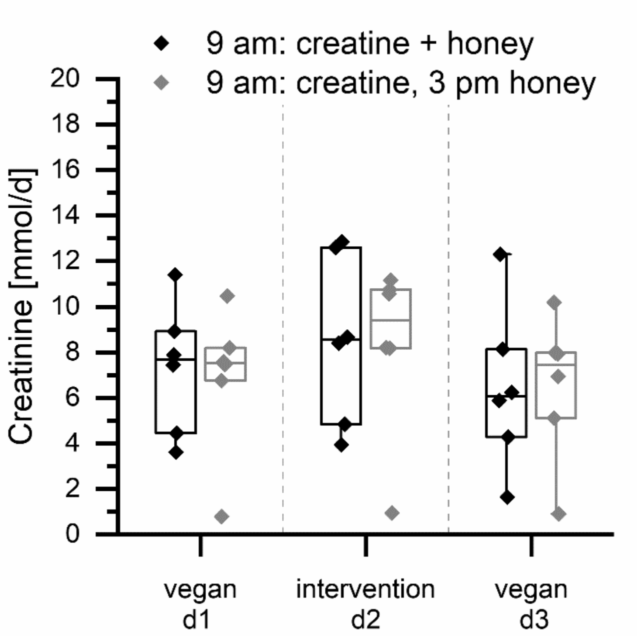

Figure S2. Daily excretion of creatinine via urine. Excretion of creatinine in 24 h urine samples of 12 volunteers during a three-day study with an intervention on day 2. Intervention: Group 1 (black): 2.5 g of creatine monohydrate + 65 g of Manuka honey (43 mg MGO) + 3 crispbreads at 9 a.m, Group 2 (grey): 2.5 g of creatine monohydrate at 9 a.m. and 65 g of Manuka honey (43 mg MGO) and 3 crispbreads at 3 p.m.
